# Editorial: Optimizing performance and injury prevention in combat sports and martial arts: methodologies for control and monitoring

**DOI:** 10.3389/fspor.2026.1824699

**Published:** 2026-03-27

**Authors:** Alex Ojeda-Aravena, Rafael Lima Kons, Eduardo Báez-San Martín, Xurxo Dopico-Calvo

**Affiliations:** 1 Departamento de Ciencias de la Actividad Física, Programa de Investigación en Deporte, sociedad y Buen Vivir., Universidad de Los Lagos, Osorno, Chile; 2 School of Kinesiology, Universidad Bernardo O'Higgins, Santiago, Chile; 3 Department of Physical Education, Faculty of Education, Universidade Federal da Bahia, Salvador, Brazil; 4 Facultad de Ciencias de la Vida, Carrera de Entrenador DeportivoUniversidad Vina del Mar, Viña del Mar, Chile; 5 Facultad de Ciencias de la Actividad Física y del Deporte, Valparaíso., Universidad de Playa Ancha, Playa Ancha, Chile; 6 Performance and Health Group, Department of Physical Education and Sport, Universidade da Coruna, A Coruña, Spain

**Keywords:** combat sports, human performance, martial arts, sport science, sports

The study of martial arts and combat sports has experienced sustained growth and progressive consolidation within the international scientific community dedicated to sport and exercise sciences (1, 2). This development has been driven both by the expansion of these disciplines within the global competitive landscape and by a substantial increase in scientific research examining their physiological, biomechanical, psychological, and technical–tactical dimensions. From a historical perspective, several combat disciplines have been included in the Olympic programme since the earliest editions of the modern Olympic Games. Fencing was part of the Olympic programme from the beginning of the modern Olympic movement, followed by wrestling and boxing, while later decades saw the inclusion of judo and taekwondo. More recently, karate was incorporated as an invited sport in the Tokyo Olympic Games. This competitive expansion has been accompanied by a steady increase in scientific publications indexed in major international databases such as Web of Science, reflecting the consolidation of this domain as a distinct field of research within sport sciences (2).

Scientific recognition of this field has also been strengthened through the institutional endorsement of leading international organizations in sport and exercise science. The National Strength and Conditioning Association (NSCA) has established a Combat Sports Special Interest Group, the American College of Sports Medicine (ACSM) increasingly incorporates combat sport research within its scientific agenda, and the European College of Sport Science (ECSS) has recently created its own group dedicated to combat sports (1). The emergence of these initiatives reflects the growing institutionalization of combat sport science within the international academic community and facilitates collaboration among researchers, practitioners, and performance specialists interested in athlete performance, health, and development.

Together, the convergence of competitive expansion, institutional recognition, and scientific development has positioned martial arts and particularly combat sports as a differentiated interdisciplinary domain within sport and exercise sciences. This field integrates physiological, biomechanical, cognitive, psychological, and technical–tactical dimensions that interact dynamically during athletic performance. From an applied perspective, combat sports are characterized by intermittent high-intensity efforts, rapid perceptual–decision processes, and complex technical–tactical interactions performed under conditions of constant uncertainty and direct confrontation with an opponent (3, 4). In such contexts, performance emerges from the integration of neuromuscular efficiency, physiological preparedness, cognitive flexibility, and emotional regulation within highly constrained temporal windows, generating an inherently multidimensional performance environment.

Beyond their competitive dimension, martial arts and combat sports also embody educational, ethical, and psychosocial values that support their application in health promotion, physical education, and long-term athlete development contexts (5, 6). Their potential to contribute to physical, psychological, and social well-being has led to their increasing integration into educational programmes and sport development initiatives. Consequently, performance optimization and injury prevention in these disciplines cannot be adequately addressed through fragmented approaches, but require integrative frameworks capable of capturing the adaptive complexity of athletes in ecological performance environments.

Within this context, this research topic was conceived to promote methodological advancement in combat sport science by encouraging approaches that integrate scientific knowledge into daily training practice and enable evidence-based monitoring, predictive modelling of performance, and sustainable athlete development. The studies included in this Research Topic illustrate the transition from predominantly descriptive analyses toward integrative and ecologically valid models designed to inform training and competition in real-world settings.

One of the key advances reflected in this collection is the adoption of multimodal approaches to performance enhancement. Traditionally, ergogenic interventions have often been examined in isolation, limiting their ecological validity. However, emerging evidence indicates that combined strategies targeting physiological, psychological, and perceptual domains produce substantially greater effects than isolated interventions (Nidhal et al.). In this regard, Jebabli et al., through a systematic review and meta-analysis including 15 studies and 1,456 participants, reported that music as a standalone intervention produced a small but significant effect on overall performance (*d* = 0.19), with stronger effects observed in psychological outcomes (*d* = 0.52). Importantly, combined interventions demonstrated markedly larger effects (*d* = 0.93), with the combination of music and caffeine producing the greatest performance enhancement (*d* = 1.24). These findings highlight that combat sport performance emerges from the interaction of multiple regulatory systems and emphasize the value of integrated preparation models.

Methodological innovation has also improved the understanding of technical–tactical behaviour during competition. Quiñones Rodríguez et al. conducted a comprehensive observational analysis of elite taekwondo athletes during the Roma 2019 World Taekwondo Grand Prix, recording 1,382 technical–tactical actions using a point, nomothetic, and multidimensional design. Through the integration of sequential analysis, polar coordinate analysis, and regression modelling, the authors identified specific scoring patterns. Direct attacks and corrections were primarily associated with single-point scoring, simultaneous counterattacks with two-point scoring, and anticipatory actions with three-point scoring. Circular techniques were frequently associated with higher scoring sequences, underscoring their strategic relevance in competitive dynamics.

Beyond athlete behaviour, the physical training environment has emerged as a relevant determinant of performance adaptation. Tu et al. examined the influence of architectural and environmental characteristics of Wushu training facilities using a mixed-methods approach involving 184 practitioners and ethnographic analysis of 24 training environments. Their results demonstrated that ceiling height, flooring material, and acoustic conditions significantly influenced psychophysiological and biomechanical outcomes. Facilities with ceilings exceeding four metres were associated with greater hip flexibility, while specialized wooden flooring enhanced cardiovascular efficiency and rigid synthetic surfaces were associated with improved explosive performance. Architectural characteristics emerged as strong predictors of flexibility (*β* = 0.73, *p* < .001) and execution speed (*β* = −0.68, *p* < .001), highlighting the role of training environments as active components of performance ecosystems.

The importance of integrated support systems was further emphasized by Yamashita et al., who investigated adaptive strategies implemented by national and professional combat sport teams during the COVID-19 pandemic. Through semi-structured interviews with performance staff, the authors identified remote training protocols, individualized load adjustments, and structured return-to-competition programmes as critical strategies for maintaining performance while minimizing injury risk. Increased concerns regarding athlete mental health also highlighted the need for psychological monitoring and multidisciplinary support structures in high-performance sport environments.

Body mass regulation remains another critical challenge in combat sports, particularly in disciplines organized by weight categories. Yáñez-Sepúlveda et al. documented the physiological and nutritional dynamics of rapid weight reduction in an elite professional boxer preparing for a world championship bout. The athlete reduced total body mass by 6.85 kg, including an acute reduction of 8.56% during the final ten days before competition. Despite maintaining a low body fat percentage and favourable somatotype characteristics, energy availability declined to critically low levels, highlighting the physiological risks associated with aggressive weight-cutting practices. These findings emphasize the importance of continuous anthropometric, physiological, and nutritional monitoring to balance competitive performance with athlete health and safety.

Collectively, the studies included in this Research Topic illustrate a paradigm shift in combat sport science, moving from isolated descriptive analyses toward integrated monitoring ecosystems incorporating physiological, technical–tactical, psychological, nutritional, and environmental variables. Advances in predictive modelling and multidimensional monitoring now enable a more precise characterization of athlete responses and performance determinants.
